# Evaluation of the effect of a resorbable membrane on the closure of palatal fistulas

**DOI:** 10.3389/fsurg.2023.1134934

**Published:** 2023-02-28

**Authors:** Yufeng Wang, Fangling Yang, Weilong Liu, Xiaofen Fan, Yong Lu

**Affiliations:** Department of Oral and Maxillofacial Surgery, Nanjing Stomatological Hospital, Medical School of Nanjing University, Nanjing, China

**Keywords:** GTR, two-layer repairs, palatal fistula repair, success rates, reduce additional damage

## Abstract

**Purpose:**

A palatal fistula following the closure of palatal clefts remains a difficult clinical complication. Surgical treatment of fistulas is often complicated, with high recurrence rates. We present our results of fistula closure augmented with GTR, a resorbable membrane designed to promote guided tissue regeneration.

**Methods:**

We reviewed the records of 75 patients operated on between 2008 and 2022 for closure of the palatal fistula. The patients included 24 who underwent fistula closure augmented with GTR and 51 who underwent fistula closure with other techniques. We reviewed the age at surgery, sex, fistula location, and outcome. Operation success was defined as an asymptomatic patient with a healed fistula on clinical examination.

**Results:**

The overall fistula closure rate was 79.1% in the GTR group and 76.5% in the non-GTR group(*p *= 0.79).

**Discussion:**

The success rate of fistula closure in the GTR group is comparable to that in the non-GTR group in this study. An additional advantage is that this procedure does not require harvesting any autologous tissue and reduces tissue damage in the long term.

## Introduction

A palatal fistula is the second most common complication post palatoplasty, after velopharyngeal insufficiency. It is characterized by a persistent fistula between the oral and nasal cavities. The incidence of palatal fistulas has been reported in different centers to range from 3% to 58%, and the recurrence rate after palatal fistula repair is generally between 25% and 60% (1, 2). According to the present study, the occurrence of a palatal fistula is related to many factors, such as the time of initial cleft palate repair, the type of cleft palate, the choice of surgical method, the surgeon's experience, and postoperative care (3).

A palatal fistula may lead to food regurgitation, velopharyngeal insufficiency, slurred speech, hearing loss, and psychological burden (4). Therefore, repairing the palatal fistula is very important and poses a considerable challenge to surgeons. Repairing the fistula requires the closure of both the nasal and oral surfaces. The nasal surface is closed mainly by the turnover of the tissue around the fistula (5). However, there is no unified standard and method for repairing the oral surface. Different methods are used according to the location and size of the fistula hole. Local, regional, and distant flaps have been well described, including rotating mucoperiosteal flaps, free flaps, tongue flaps, and buccal musculomucosal flaps. In addition, autografts of the mucosa, fat, cartilage, bone, fascia, muscle, and dermis have also been widely reported (6–11). With the rapid development of bioengineering, some exogenous synthetic materials have been applied, such as acellular dermal matrices and collagen membranes (12–14). Among them, absorbable collagen membranes are widely used as tissue barriers in guided tissue regeneration (15). In palatal fistula repair surgery, the application of collagen membranes can not only reduce tension at the palatal fistula site but also provide a scaffold that facilitates and stimulates the growth of tissues, revascularization, and mucosal epithelialization. Most importantly, acting as a tissue barrier, the collagen membranes can prevent the epithelium around the fistula from growing into the nasal cavity to form a new fistula (16).

In current studies, collagen membrane-assisted palatal fistula repair usually adopts a three-layer repair method, consisting of the nasal mucosa, an interpositional collagen membrane, and a rotational palatal mucoperiosteal flap (4). Although various studies have shown that this method can significantly improve the success rate of palatal fistula repair, it does not reduce tissue damage compared with the traditional two-layer method (the nasal mucosa and rotational palatal flaps). The additional incision in the preparation of the mucoperiosteal flap may cause bleeding, inflammation, and other complications, which are not conducive to healing in the short term. It may be detrimental to normal maxillary growth and development in the long term due to increased scarring (17). Therefore, the authors proposed to improve the original three-layer repair method, only covering the nasal mucosa with a layer of collagen membrane to close the oral side, without the need to prepare a mucoperiosteal flap to cover the oral cavity as a third layer, to achieve the purpose of closing the palatal fistula and reduce additional incision damage. In this paper, the authors present our experience of fistula closure over a 14-year period, specifically comparing the success rates of those two-layer repairs augmented with GTR (Bote BioTech. Co., Ltd., Fujian, China) with those closed by traditional methods.

## Materials and methods

Over a 14-year period, from 2008 to 2022, 75 patients (54 men and 21 women; age: 1–50 years; median: 11 years) underwent palatal fistula closure. The senior author first introduced the use of GTR (Bote BioTech. Co., Ltd., Fujian, China) in 2017; prior to this, palatal fistulas were closed using only local flaps.

The standard surgical method used for fistula management was based on the known technique. The surgery was performed under general anesthesia. Prophylactic antibiotics were administered before surgery. The incisional lines were designed with blue methylene amine. The length between the orifice and the incision was 2 mm longer than half the size of the defect, so we could turn over flaps that were raised and sutured to form the nasal layer. After the injection of a local anesthetic solution containing adrenaline to facilitate dissection and decrease the risk of local bleeding, the incisions were placed surrounding the fistula in a circular manner, and the fistula's mucosa was reverted toward the nasal cavity. In the non-GTR group, according to the traditional palatoplasty technique, two full-thickness palatal flaps were prepared, one on each side. The dissected mucosal edges of the fistula were sutured using a 5-0 resorbable suturing material toward the nasal cavity to create a nasal mucosal layer bridging the defect. In this way, the raw surface was exposed toward the oral side. In the GTR group, a resorbable collagen membrane (Bote BioTech. Co., Ltd., Fujian, China) was applied over the already reconstructed nasal layer. This ensured the defect was covered with a second layer. In the non-GTR group, the second layer, or the oral layer, was covered by rotating and advancing the already prepared palatal flaps without any tension to rebuild the oral mucosa with a 3-0 resorbable suturing material. Large fistulas requiring three-layer repair were not included in this study. Postoperative antibiotics were prescribed, and the patients remained on a liquid and cold diet for 2 weeks. A successful operation was defined as an asymptomatic patient with a healed fistula on clinical examination. A chi-squared test was used to statistically compare the success rates in fistula closure between the two groups, with a *p*-value of <0.05 denoting a statistically significant result.

## Results

The study included 75 fistula operations. The surgeries comprised 24 fistula closures augmented with GTR, which were compared with the remaining 51 repairs, which used local tissue only.

Table 1 shows the main characteristics of the two groups. Near-equal gender distribution and median age at initial surgery were observed in the GTR group, and the non-GTR group included 40 men and 11 women. The median age at initial surgery was 17.5 months and 18 months in the GTR group and non-GTR group, respectively. Most patients with palatal fistulas are referred from out-of-town hospitals. The traditional Langenbeck method or the two-flap method was used in primary cleft palate repair. Cleft palate repair using muscle reconstruction or the Furlow method is mainly concentrated in a few large medical schools. In our hospital, the Sommerlad method is used to repair the cleft palate. The median age for fistula closure was 9.3 years and 12.5 years in the GTR group and non-GTR group, respectively. Table 2 shows the distribution of the fistula in the two groups, classified according to the Pittsburgh classification. As shown in Table 2, type III and type IV, located at the junction of the hard/soft palate and hard palate, constituted the vast majority in both groups. The size of the palatal fistula was subdivided into small (1–2 mm), medium (3–5 mm), and large (>5 mm). Table 3 shows the size of the fistula in the GTR group and non-GTR group. There was no significant difference in the size of the fistula between the two groups.

**Table 1:** Main characteristics of the two groups of fistula repairs.

Method of closure	Fistulas augmented with GTR	Fistulas repaired using local tissue only
Number in each cohort	24	51
Male (M)/female (F) distribution	M = 14 F = 10	M = 40 F = 11
Median age at fistula closure	9.3 years	12.5 years
Median age at initial surgery	17.5 months	18 months

**Table 2:** The distribution of the fistula in the two groups according to the Pittsburgh classification.

Pittsburgh classification	GTR group	Non-GTR group
I-Uvular	0 (0%)	1 (2%)
II-Soft palate	6 (25%)	9 (17.6%)
III-Junction hard/soft palate	11 (45.8%)	14 (27.4%)
IV-Hard palate	6 (25%)	15 (29.4%)
V-Junction primary/secondary palate	1 (4.2%)	2 (4%)
VI-Lingual-alveolar	0 (0%)	5 (9.8%)
VII-Labial-alveolar	0 (0%)	5 (9.8%)

**Table 3:** The size of the fistula in the two groups.

	Small	Medium	Large	
GTR group	3	8	13	
Non-GTR group	5	17	29	*p* = 0.936
Total	8	25	42	

**Figure 1:**
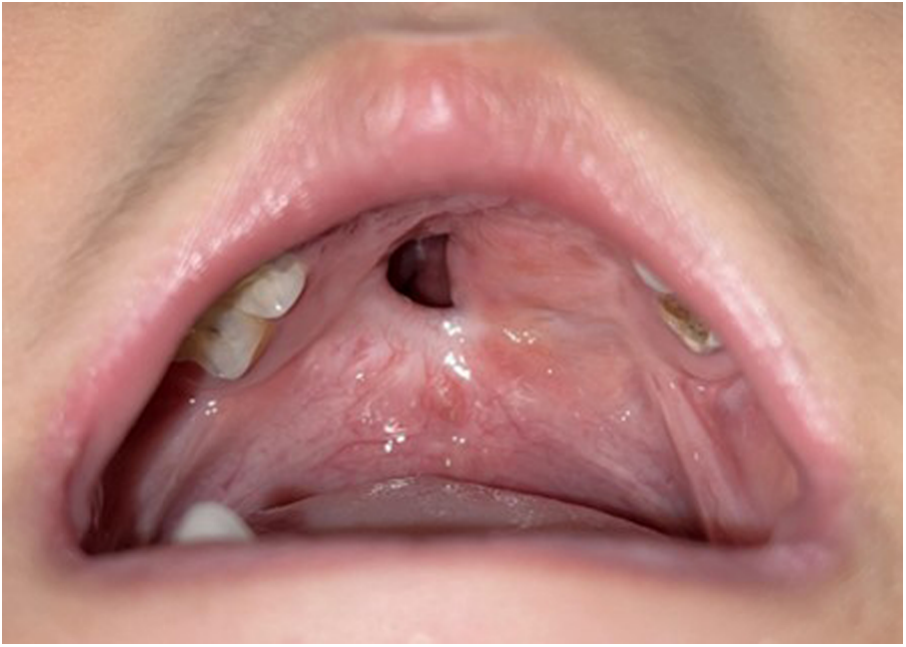
Preoperative appearance of a mid-hard palate fistula.

**Figure 2:**
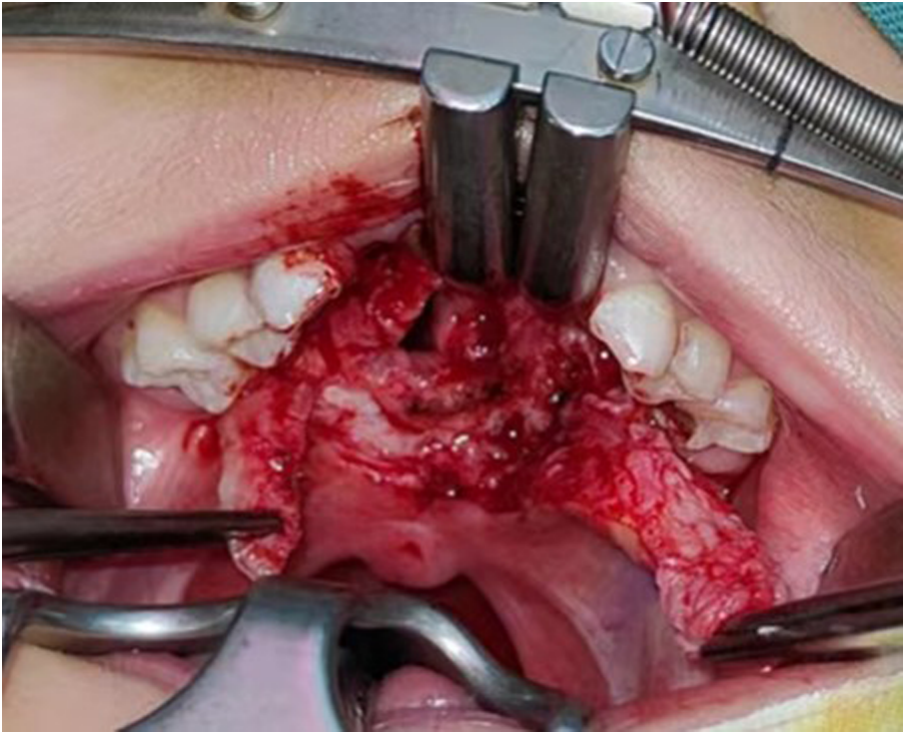
Preparation of two bridge flaps.

**Figure 3:**
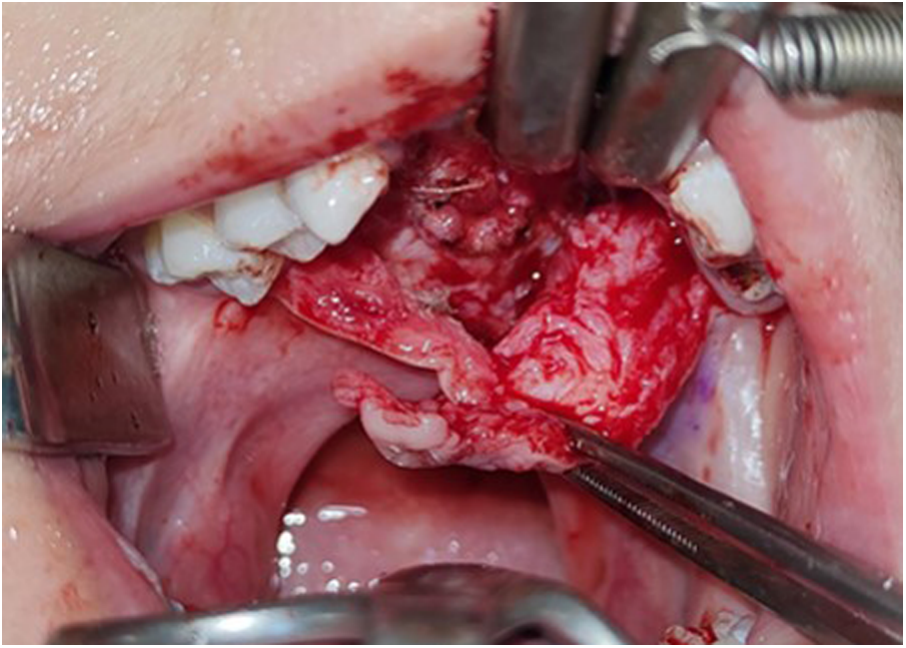
The nasal mucosa is closed as the first layer.

**Figure 4:**
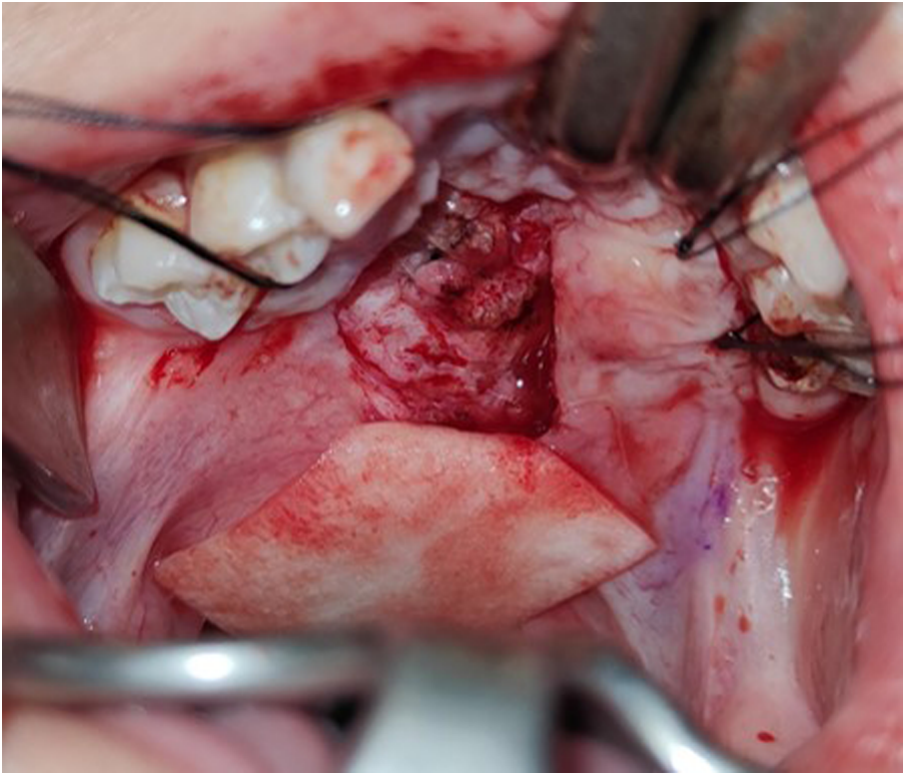
GTR membrane is inserted as the second layer above the nasal mucosa.

**Figure 5:**
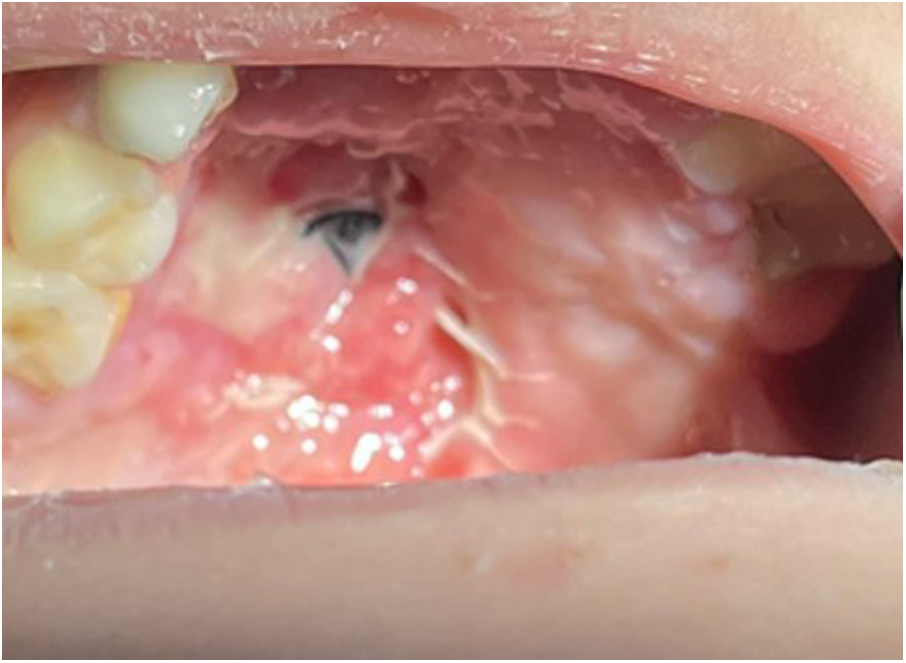
Postoperative appearance 2 weeks after the palatal fistula closure operation.

A successful operation was defined as an asymptomatic patient with a healed fistula on clinical examination. Figures 1–5 show a typical fistula and the outcome of using GTR as an oral layer. In the GTR group, the shortest follow-up time was 3 months and the longest follow-up time was 67 months. In the non-GTR group, the shortest follow-up time was 2 months and the longest follow-up time was 171 months. The overall success rate of fistula repair in the GTR group was 79.1%, while the success rate prior to the introduction of non-GTR was 76.5% (Table 4), with no significant difference. Compared with traditional palatal fistula repair surgery, two-layered repair with the aid of a resorbable collagen membrane for the palatal fistula did not prolong the operation significantly. No membrane-related adverse reaction was observed. There was no evidence of infection, rejection, or dehiscence. There was no fistula recurrence.

**Table 4:** The results of cleft palate fistula closure.

	Success	Failure	Success rate	
GTR group	19	5	79.1	
Non-GTR group	39	12	76.5	*p* = 0.79
Total	58	17	77.3	

## Discussion

A palatal fistula is a common complication after cleft palate repair, usually occurring in the anterior part of the hard palate, the junction of the hard and soft palate, the palatal sag, and the anterior part of the soft palate (18–20). A large fistula can lead to a series of problems such as language dysfunction, hearing impairment, poor oral and nasal hygiene, and psychological abnormalities. The key to repairing a palatal fistula is to design a healthy and viable tissue flap to completely cover the fistula hole and reconstruct the anatomic integrity of the nasal cavity and oral cavity. To date, there is no unified and fixed surgical method for treating palatal fistula. Flexible and variable surgical methods are adopted to comprehensively treat the palatal fistula according to different types of fistula holes. The most common flap is a local mucosal flap, which uses overlapping mucosal flaps to cover the fistula. However, this kind of local mucoperiosteal flap repair will cause the bone surface of the donor site to be exposed, which is easily affected by environmental factors. The repair of the bare bone surface finally generates scar healing through the regeneration of granulation tissue, which destroys the blood supply of the jaw and affects the growth center of the jaw, resulting in facial jaw deformity. Minimizing the inhibitory effect of the exposed bone trauma after palatal fistula repair on the growth and development of the maxilla is still a significant problem (21).

Over the last 30 years, guided tissue regeneration therapy has been extensively practiced in dentistry with the rapid development of tissue engineering and biological materials. The application of interpositional materials not only reduces tension at the fistula site but also provides a scaffold and barrier that stimulates tissue growth and prevents epithelium around the fistula from growing into the nasal cavity to form a new fistula. Moreover, the resorbable allograft membrane does not create additional donor sites for the body, thus reducing tissue damage that may lead to jaw deformity. As a resorbable membrane, GTR has good long-term biocompatibility and safety. In this study, the success rate in the GTR group was 79.1%, not significantly different from the traditional method.

Although this is a retrospective study with some limitations, this study is based on a single surgeon's experience, and the results were followed up for 14 years. Of course, due to the use of GTR being almost in its late stages, there could be a “learning curve” over time, but this study still provides a certain reference value for the surgical treatment of palatal fistulas.

## Data Availability

The original contributions presented in the study are included in the article/Supplementary Material, further inquiries can be directed to the corresponding author.

## References

[1] Smarius BJ Breugem CC. Use of early hard palate closure using a vomer flap in cleft lip and palate patients. J. Cranio Maxillo Fac Surg. (2016) 44:912–8. 10.1016/j.jcms.2016.05.011 27263756

[2] Schultz RC. Management and timing of cleft palate fistula repair. Plast Reconstr Surg. (1986) 78:739. 10.1097/00006534-198678060-00004 3538077

[3] Aziz SR Rhee ST Redai I. Cleft surgery in rural Bangladesh: reflections and experiences. J Oral Maxillofac Surg. (2009) 67(8):1581–8. 10.1016/j.joms.2008.11.021 19615567

[4] Alonsoa V Sanchez- Abuina A Duranb C Gomeza O Migueza L Molina ME. Three-layered repair with a collagen membrane and a mucosal rotational flap reinforced with fibrine for palatal fistula closure in children. Int J Pediatr Otorhinolaryngol (2019) 127:109679. 10.1016/j.ijporl.2019.109679 31536855

[5] Wilhelmi BJ Appelt EA Hill L Blackwell SJ. Palatal fistulas: rare with the two flap palatoplasty repair. Plast Reconstr Surg. (2001) 107(2):315–8. 10.1097/00006534-200102000-00002 11214043

[6] Honnebier MB Johnson DS Parsa AA Dorian A Parsa FD. Closure of palatal fistula with a local mucoperiosteal flap lined with buccal mucosal graft. Cleft Palate Craniofac J. (2000) 37(2):127–9. 10.1597/1545-1569_2000_037_0127_copfwa_2.3.co_2 10749052

[7] Mosaad AA. V-Y two-layer for oronasal fistula of hard palate. Int J Pediatr Otorhinolaryngol. (2010) 74:1054–7. 10.1016/j.ijporl.2010.06.003 20591506

[8] Mosaad AA Wael AN Hassan EH Ahmed H Badawi K. Closure of anterior post-palatoplasty fistula using superior lip myomucosal flap. Int J Pediatr Otorhinolaryngol. (2008) 72:571–4. 10.1016/j.ijporl.2008.01.009 18295354

[9] Mosaad AA. The use of buccal flap in the closure of posterior post-palatoplasty fistula. Int J Pediatr Otorhinolaryngol. (2008) 72:1657–61. 10.1016/j.ijporl.2008.07.020 18814922

[10] Lahiri AK Richard B. Superiorly based facial artery musculomucosal flap for large anterior palatal fistulae in cleft. Cleft Palate Craniofac J. (2007) 44(5):523–7. 10.1597/06-164.1 17760492

[11] Assuncao AG. The design of tongue flap for the closure of palatal fistula. Plast Reconstr Surg. (1993) 91(5):806–10. 10.1097/00006534-199304001-00008 8460182

[12] Jeffery SLA Boorman JG Dive DC. Use of cartilage grafts for closure of cleft palate fistulae. Br J Plast Surg. (2000) 53:551–4. 10.1054/bjps.2000.3411 11000068

[13] Steele MH Seagle MB. Palatal fistula repair using acellular dermal matrix: the university of Florida experience. Ann Plast Surg (2006) 56(1):50–3. 10.1097/01.sap.0000185469.80256.9e 16374096

[14] Zhang B Li J Sarma D Zhang F Chen J. The use of heterogeneous acellular dermal matrix in the closure of hard palatal fistula. Int J Pediatr Otorhinolaryngol. (2014) 78(1):75–8. 10.1016/j.ijporl.2013.10.053 24290949

[15] Sader R Seitz O Kuttenberger J. Resorbable collagen membrane in surgical repair of fistula following palatoplasty in nonsyndromic cleft palate. Int J Oral Maxillofac Surg. (2010) 39(5):497e9. 10.1016/j.ijom.2010.02.012 20227244

[16] Atherton DD Boorman JG. Use of a purified collagen membrane to aid closure of palatal fistulae. J Plast Reconstr Aesthet Surg. (2016) 69(7):10037. 10.1016/j.bjps.2016.02.009 27039219

[17] Li Y Wu M Yang C Tsauo C Li C Liu R Evaluation of fistula rates in three cleft palate techniques without relaxing incisions. J Cranio Maxillo Fac Surg. (2021) 49(6):456461. 10.1016/j.jcms.2021.01.022 33581960

[18] Parwaz MA Sharma RK Parashar A Nanda V Biswas G Makkar S. Width of cleft palate and postoperative palatal fistula: do they correlate? J Plast Reconstr Aesthet Surg. (2009) 62:1559–63. 10.1016/j.bjps.2008.05.048 18838320

[19] Landheer JA Breugem CC van der Molen AB. Fistula incidence and predictors of fistula occurrence after cleft palate repair: two-stage closure versus one-stage closure. Cleft Palate Craniofac J. (2010) 47:623–30. 10.1597/09-069 21039279

[20] Andersson EM Sandvik L Semb G Abyholm F. Palatal fistulas after primary repair of clefts of the secondary palate. Scand J Plast Reconstr Surg Hand Surg. (2008) 42:296–9. 10.1080/02844310802299676 18991171

[21] Reddy RR Gosla Reddy S Vaidhyanathan A Berge SJ Kuijpers-Jagtman AM. Maxillofacial growth and speech outcome after one-stage or twostage palatoplasty in unilateral cleft lip and palate. A Systematic Review. J Cranio Maxill Surg. (2017) 45(6):995–e1003. 10.1016/j.jcms.2017.03.006 28427835

